# Effects of alternative and successive resistance training methods on the muscle fatigue of concentric and eccentric contractions in healthy male individuals

**DOI:** 10.3389/fspor.2025.1640202

**Published:** 2025-11-12

**Authors:** Masafumi Kadota, Masatoshi Nakamura, Riku Yoshida, Kosuke Takeuchi

**Affiliations:** 1 Department of Physical Therapy, Kobe International University, Kobe-shi, Hyogo, Japan; 2 Amarneiss Garden, Amagasaki-shi, Hyogo, Japan; 3 Faculty of Rehabilitation Sciences, Nishi Kyushu University, Kanzaki-cho, Saga, Japan; 4 Department of Rehabilitation Medicine, Maniwa Orthopedics Clinic, Niigata-shi, Niigata, Japan

**Keywords:** quadriceps, hamstrings, training order, rest interval, traditional training, paired set training

## Abstract

**Introduction:**

The effects of alternative and successive training on muscle fatigue profiles of concentric (CON) and eccentric (ECC) contractions were examined.

**Methods:**

Seventeen untrained men performed alternative and successive training with maximum isokinetic muscle contractions. In alternative training, three sets of knee flexion and extension exercises were alternatively performed with a 60-s rest interval. Successive training completed three sets of knee flexion exercises followed by three sets of knee extension exercises with a 60-s rest interval. Muscle strength and training volume were measured.

**Results and Discussions:**

Knee flexion muscle strength did not change in either the CON (*p* = 0.148) or ECC phases (*p* = 0.073). Knee extension muscle strength decreased in the CON phase (*p* = 0.004), but it did not change in the ECC phase (*p* = 0.415). The training volume of knee flexion decreased with each set in the CON phase (*p* < 0.01), but it decreased in Set 3 in the ECC phase (*p* < 0.01). The training volume of knee extension decreased with each set in the CON phase (*p* < 0.01), but it showed no change between sets in the ECC phase (*p* > 0.05). Alternative training had a lower rate of the change of decrease in training volume for knee extension than did successive training (*p* < 0.05). The total training volume was higher in alternative training than in successive training (*p* < 0.05). These results indicated that the ECC phase had less fatiguability than the CON phase, regardless of training methods. Moreover, the alternative training used in this study resulted in less muscle fatigue in the quadriceps and a larger total training volume than the successive training.

## 1 Introduction

Resistance training consists of concentric contractions (CON), eccentric contractions (ECC), or a combination of both contractions (CON + ECC) (1–5). Many previous studies have compared the effects of CON and ECC (2, 3, 6, 7). ECC shows greater muscle hypertrophy and strength gains than CON, which may be partly explained by the greater training volume generated by ECC (2, 3, 6, 7). In conventional resistance training, trained muscles alternately perform concentric and eccentric contractions (8–10). Yoshida et al. (4) examined the immediate effects of 30 repetitions of maximum isokinetic elbow flexion exercises with CON + ECC on peak torque during the repetitions. They reported that the elbow flexion torque during training was greater in the ECC phase than in the CON phase and that the rate of decrease in elbow flexion torque was lower in the ECC phase compared to the CON phase. Therefore, the CON and ECC phases of CON + ECC exhibit different muscle strength and muscle fatigue characteristics.

ECC has less fatiguability compared to CON (1, 4, 11, 12). ECC recruits fewer motor units than CON when the muscle contraction (13) and muscle strength of the ECC involve elastic forces of connective tissues such as titin (13), which result in a lower energy cost of ECC compared to CON (1). Yoshida et al. (4) investigated the muscle fatigue of maximum isokinetic contractions using two variables: rate of torque decrease during training and isometric maximum muscle strength before and after training. They reported that the rate of torque decrease during training was lower in the ECC, but there was no significant difference in the isometric muscle strength before and immediately after training between CON and ECC. These data suggested that characteristics of muscle fatigue in CON and ECC could be different during and after resistance training. However, since ECC changes the optimal angle of maximum muscle strength (14), it is possible that the muscle fatigue of ECC cannot be accurately determined using isometric contractions. To our best knowledge, no study has examined the differences in muscle fatigue between CON and ECC during and after resistance training using an isokinetic muscle contraction. Muscle fatigue from resistance training is related to training volume (15–17), and thus, it is important to accurately examine the muscle fatigue of CON and ECC.

Resistance training generally consists of multiple training exercises (15, 18–20). When performing multiple training exercises, alternative and successive training methods are widely used (9, 20–22). In alternative training, sets of different exercises are performed aternately, whereas in successive training, all sets of one type of exercise are completed before proceeding to the next exercise. When the agonist muscle is trained, the antagonist muscle is not activated (14). In the alternating training method of agonist and antagonist muscles, the antagonist muscles and inter-set rest intervals are the rest time before the next agonist muscle training, but in the successive training method, only the inter-set rest interval is the rest time. Therefore, even if the alternative and successive training of agonist and antagonist muscles are performed with the same number of repetitions, sets, and inter-set rest intervals, the rest periods for each muscle in the alternative training are longer than successive training, resulting in greater training volume, which was calculated from the torque-time curve during training for alternative training (22, 23). The influence of the training interval period is greater for CON than for ECC (1). Shibata et al. (1) compared the effects of different inter-repetition periods (3 and 6 s) on repetitions to failure between ECC and CON. They found that failure to repetitions did not change in ECC but increased in CON (1.4–1.5 times greater in 6 s than in 3 s). Therefore, the difference between alternating and successive training methods may affect muscle fatigability, especially in CON. However, the effects of training methods on muscle fatigue of CON and ECC are unclear. Because muscle fatigue is directly related to training volume (17, 24), it is necessary to investigate the effects of resistance training methods on muscle fatigue in CON and ECC to develop effective resistance training methods.

Therefore, this study aimed to examine the effects of alternative and successive training on muscle fatigue of the CON and ECC phases of CON + ECC. The hypothesis was that the ECC phase has less fatiguability compared to the CON phase based on previous studies (1, 4, 11, 12). Moreover, it was hypothesized that the difference in the training methods may have a greater effect on the muscle fatigue of the CON phase than that of the ECC phase.

## 2 Materials and methods

### 2.1 Experimental approach

**Figure 1:**
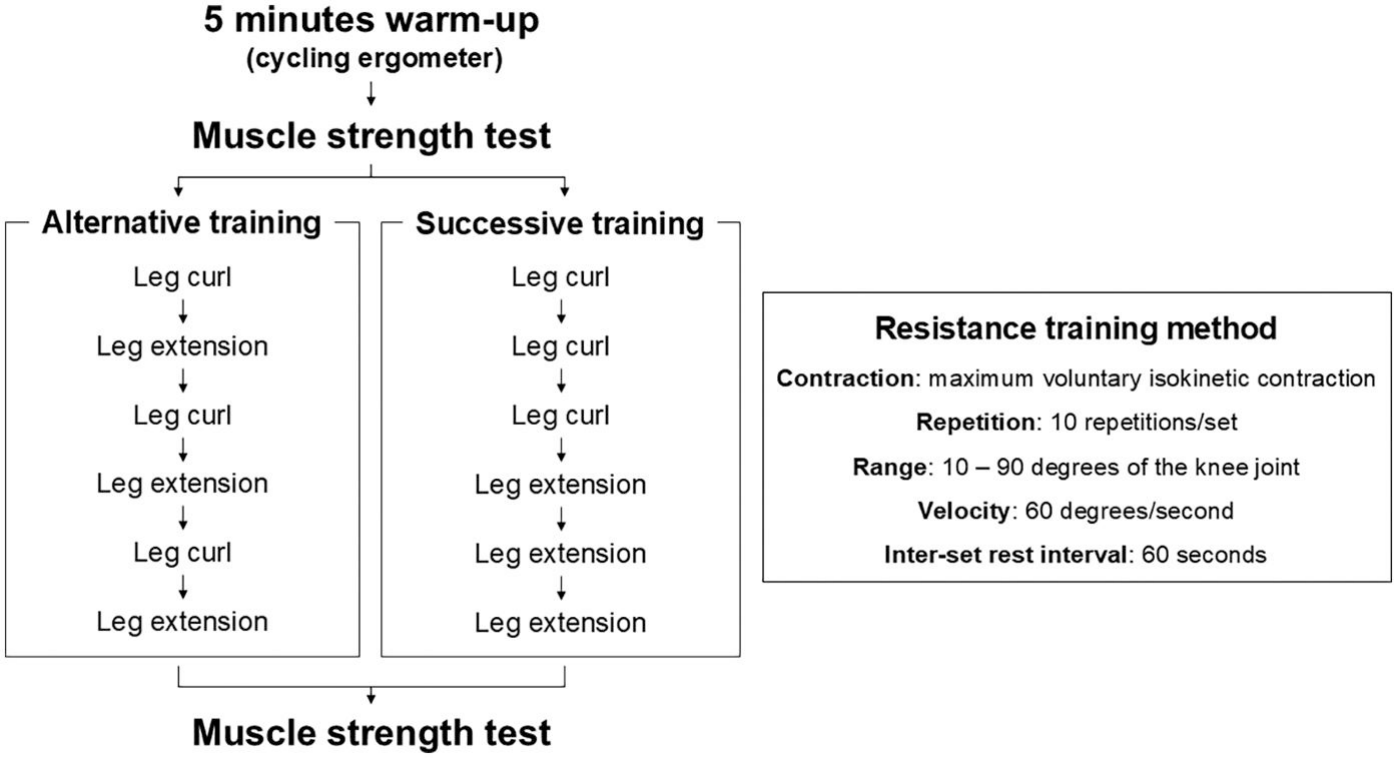
Experiment protocol.

In the present study, a randomized, repeated crossover design was used. The knee extensors and flexors of the dominant limb (ball kicking preferred) were used (25). The participants underwent two different resistance training interventions (alternative and successive training) with an interval of 2 weeks between visits. The experimental procedure was as follows: 5 min warm-up with a cycling ergometer (60 W, 120 bpm), familiarization session, pre-strength measurement, resistance training interventions, and post-strength measurement (Figure 1). In the familiarization session, the participants practiced voluntary isokinetic contractions of the knee extension and flexion with light loads (23). To assess muscle fatigue, muscle strength before and immediately after resistance training interventions and training volume during training were evaluated. The experiment was performed in a university laboratory, where the temperature was maintained at 24 °C.

### 2.2 Participants

Seventeen healthy and untrained male university students participated in this study (20.5 ± 0.3 years, 170.1 ± 5.2 cm, 63.3 ± 10.1 kg). Participants with a history of pathology in the lower limb within 6 months were excluded. The sample size of training volume was calculated with a repeated measures within-between interaction, power of 80%, alpha error of 0.05, and effect size of 0.25 (middle) using G*Power 3.1 software (Heinrich Heine University, Düsseldorf, Germany), and the results showed that the requisite number of participants for this study was 17 participants. All participants were informed of the requirements and risks associated with their involvement in this study and signed a written informed consent document. The study was performed in accordance with the Declaration of Helsinki (1964). This study was approved by the Ethics Committee (Procedure #G2023-186).

### 2.3 Muscle strength test

Muscle strength was measured using an isokinetic dynamometer machine (CYBEX NORM, Humac, California, USA). The participants sat on the dynamometer, and the angle between the backrest and the seat was set at 100 degrees (23). The trunk and thigh of the dominant leg were firmly secured with straps. The knee joint was aligned with the axis of the rotation of the isokinetic dynamometer machine. The lever arm attachment was placed just proximal to the malleolus medialis and stabilized with straps. The range of motion of the knee joint during the muscle strength measurement was 10–90° knee flexion, and the angular velocity was 60 °/s. Muscle strength measurements were performed in the following order: knee flexion CON, knee flexion ECC, knee extension CON, and knee extension ECC. The participants were instructed to perform maximum voluntary isokinetic concentric and eccentric contractions. The muscle strength test of each muscle was performed three times, and the greatest value was used for the analyses.

### 2.4 Resistance training

The participants were positioned on the isokinetic dynamometer machine in the same fashion as the muscle strength measurement. Resistance training interventions of knee flexion and knee extension exercises were performed for three sets of ten repetitions each. The range of motion of each training was 10–90° knee flexion, and the angular velocity was 60 °/s. The participants were instructed to perform maximum voluntary isokinetic concentric and eccentric contractions in each repetition. Participants rested in a seated position on the dynamometer machine during the 60 s of inter-set rest intervals.

#### 2.4.1 Alternative training

The knee flexion exercise and knee extension exercise were performed alternatively with 60 s of inter-set rest intervals between each exercise.

#### 2.4.2 Successive training

First, three sets of knee flexion exercises were performed. Thereafter, three sets of knee extension exercises were performed. The inter-set rest intervals between each exercise were set for 60 s.

### 2.5 Training volume

**Figure 2:**
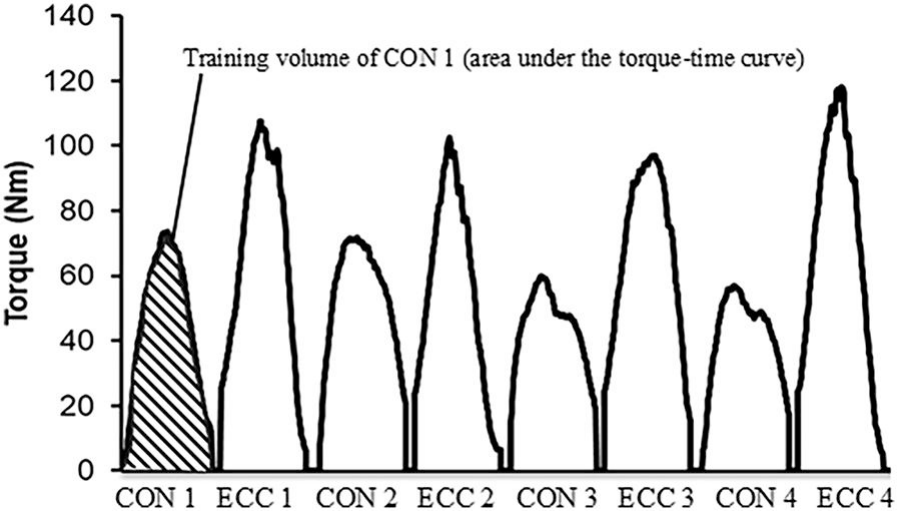
Calculation of training volume (area under the torque angle curve). CON: concentric contraction. ECC: eccentric contraction.

Knee flexion and extension torques during resistance training interventions were recorded with the dynamometer machine. Training volume was defined as the areas under the torque-time curve of each contraction, and it was calculated by integrating the torque (Figure 2) (23). Training volume per set (10 repetitions) and total training volume (30 repetitions) were calculated separately for the CON phase and ECC phase (4, 23).

### 2.6 Statistical analysis

The Shapiro–Wilk test was used to assess the normal distribution and all data. All data were normally distributed. For the muscle strength data, a repeated three-way ANOVA (time [pre vs. post] × interventions [alternative training vs. successive training] × contractions [CON phase vs. ECC phase]) were used. For the training volume data, repeated three-way ANOVA (time [set 1 vs. set 2 vs. set 3] × interventions [alternative training vs. successive training] × contractions [CON phase vs. ECC phase]) were used. For total training volume data, a repeated two-way measures ANOVA (interventions [alternative training vs. successive training] × contractions [CON phase vs. ECC phase]) was used. If a significance was detected, *post hoc* analyses using Bonferroni's test were performed. Partial *η*^2^ values were reported to reflect the magnitude of the differences for each treatment (small = 0.01, medium = 0.06, and large = 0.14) (26). To examine the effect of training methods on the muscle strength and training volume in the CON and ECC phases, the relative change in muscle strength (post/pre ratio) and training volume (set 3/set 1 ratio) in the CON and ECC phases were analyzed using a paired *t*-test. The analyses were performed using SPSS Version 25 (SPSS, Inc., Chicago, IL, USA). Differences were considered statistically significant at an alpha of 0.05.

## 3 Results

### 3.1 Muscle strength

For the muscle strength of the knee flexion, there was no significant three-way interaction (*p* = 0.093, partial *η*^2^ = 0.167) (Table 1). There were no significant two-way interactions between interventions and time (*p* = 0.199, partial *η*^2^ = 0.101) or interventions and contractions (*p* = 0.727, partial *η*^2^ = 0.008), but there was a significant two-way interaction between time and contractions (*p* = 0.003, partial *η*^2^ = 0.440). The CON phase was lower than the ECC phase for pre (*p* = 0.001) and post values (*p* < 0.001). There was no significant difference between pre- and post-values in the CON (*p* = 0.148) and ECC phases (*p* = 0.073).

**Table 1:** Muscle strength data.

	Alternative training		Successive training	
	Pre (Nm)	Post (Nm)	Pre (Nm)	Post (Nm)
Knee flexion				
CON phase	75.0 ± 12.2	69.1 ± 9.2	69.8 ± 12.9	70.8 ± 15.8
ECC phase	84.4 ± 20.2*	88.8 ± 15.7*	83.7 ± 20.4*	87.5 ± 20.8*
Knee extension				
CON phase	125.0 ± 29.6	99.9 ± 26.9^†^	114.0 ± 27.0	100.5 ± 18.6^†^
ECC phase	135.5 ± 36.4*	139.1 ± 37.2*	129.7 ± 27.9*	134.7 ± 24.3*

CON, concentric contraction; ECC, eccentric contraction. Values were described as mean ± standard deviation.

* *p* < 0.05 vs. CON phase.

† *p* < 0.05 vs. pre-value.

For the muscle strength of the knee extension, there was no significant three-way interaction (*p* = 0.142, partial *η*^2^ = 0.130) (Table 1). There were no significant two-way interactions between interventions and time (*p* = 0.348, partial *η*^2^ = 0.055) or interventions and contractions (*p* = 0.986, partial *η*^2^ < 0.001), but there was a significant two-way interaction between time and contractions (*p* < 0.001, partial *η*^2^ = 0.665). The CON phase was lower than the ECC phase for pre (*p* = 0.001) and post values (*p* < 0.001). There was a significant difference between pre- and post-values in the CON phase (*p* = 0.004) but not in the ECC phase (*p* = 0.415).

### 3.2 Training volume

For the training volume of the knee flexion, there was no significant three-way interaction (*p* = 0.928, partial *η*^2^ = 0.005) (Table 2). There were no significant two-way interactions between interventions and time (*p* = 0.110, partial *η*^2^ = 0.129) or interventions and contractions (*p* = 0.481, partial *η*^2^ = 0.032), but there was a significant two-way interaction between time and contractions (*p* = 0.011, partial *η*^2^ = 0.245). The CON phase was lower than the ECC phase in Set 1 (*p* = 0.007), Set 2 (*p* = 0.001), and Set 3 (*p* = 0.001). In the CON phase, Set 1 was significantly higher than Set 2 (*p* < 0.001) and Set 3 (*p* < 0.001), and Set 2 was significantly higher than Set 3 (*p* = 0.001). In the ECC phase, there was no significant difference between Set 1 and Set 2 (*p* = 0.840), but Set 1 (*p* = 0.029) and Set 2 (*p* = 0.001) were significantly higher than Set 3.

**Table 2:** Training volume of the knee flexion.

	Alternative training			Successive training		
	Set 1 (Nm)	Set 2 (Nm)	Set 3 (Nm)	Set 1 (Nm)	Set 2 (Nm)	Set 3 (Nm)
CON phase	597.9 ± 103.4	565.0 ± 90.8^†^	526.7 ± 92.9^†^^,^^‡^	562.7 ± 91.8	496.9 ± 89.3^†^	472.9 ± 74.3^†^^,^^‡^
ECC phase	685.4 ± 183.6*	691.6 ± 183.8*	650.4 ± 156.8^*^^,^^†^^,^^‡^	636.1 ± 162.4*	601.9 ± 144.7*	576.2 ± 151.2^*^^,^^†^^,^^‡^

CON, concentric contraction; ECC, eccentric contraction. Values were described as mean ± standard deviation.

* *p* < 0.05 vs. CON phase.

† *p* < 0.05 vs. Set 1.

‡ *p* < 0.05 vs. Set 2.

For the training volume of the knee extension, there was no significant three-way interaction (*p* = 0.782, partial *η*^2^ = 0.015) (Table 3). There were no significant two-way interactions between interventions and contractions (*p* = 0.052, partial *η*^2^ = 0.216) but there was a significant two-way interaction between interventions and time (*p* = 0.041, partial *η*^2^ = 0.181) and time and contractions (*p* = 0.030, partial *η*^2^ = 0.197). Alternative training was significantly higher than successive training in Set 2 (*p* = 0.002) and Set 3 (*p* < 0.001) but not Set 1 (*p* = 0.157). In alternative training, there was no significant difference between sets (Set 1 vs. Set 2; *p* = 0.890, Set 2 vs. Set 3; *p* = 0.638, Set 1 vs. Set 3; *p* = 0.542), while there was a significant difference in successive training between sets (Set 1 vs. Set 2; *p* = 0.025, Set 2 vs. Set 3; *p* = 0.006, Set 1 vs. Set 3; *p* = 0.001). The CON phase was significantly lower than the ECC phase in Set 2 (*p* = 0.030) and set 3 (*p* = 0.009) but not in Set 1 (*p* = 0.165). In the CON phase, there was a significant difference between sets (Set 1 vs. Set 2; *p* = 0.003, Set 2 vs. Set 3; *p* = 0.011, Set 1 vs. Set 3; *p* = 0.002), while there was no significant difference in the ECC phase between sets (Set 1 vs. Set 2; *p* > 0.99, Set 2 vs. Set 3; *p* = 0.502, Set 1 vs. Set 3; *p* = 0.334).

**Table 3:** Training volume of the knee extension.

	Alternative training			Successive training		
	Set 1 (Nm)	Set 2 (Nm)	Set 3 (Nm)	Set 1 (Nm)	Set 2 (Nm)^§^^,^^#^	Set 3 (Nm)^§^^,^^#^^,^^¶^
CON phase	877.5 ± 248.5	826.6 ± 190.9^†^	781.7 ± 173.6^†^^,^^‡^	843.3 ± 225.0	744.9 ± 172.2^†^	683.3 ± 143.5^†^^,^^‡^
ECC phase	984.5 ± 280.6	992.5 ± 304.1*	976.5 ± 278.3*	896.4 ± 197.2	834.7 ± 267.1*	786.2 ± 233.7*

CON, concentric contraction; ECC, eccentric contraction. Values were described as mean ± standard deviation.

* *p* < 0.05 vs. CON phase.

† *p* < 0.05 vs. Set 1 of CON phase.

‡ *p* < 0.05 vs. Set 2 of CON phase.

§ *p* < 0.05 vs. alternative training at the same set.

# *p* < 0.05 vs. set 1 in the successive training.

¶ *p* < 0.05 vs. set 2 in the successive training.

For the total training volume, alternative training (9,157 ± 1,859 Nm) was significantly higher than successive training (8,118 ± 1,331 Nm) (*p* = 0.005, *d* = 0.795).

### 3.3 Relative change in muscle strength and training volume

In the relative change in the muscle strength of knee flexion (CON phase of *p* = 0.106 and ECC phase *p* = 0.726) and knee extension (CON phase of *p* = 0.132 and ECC phase *p* = 0.940), there was no significant difference between alternative and successive training (Table 4). In the relative change in the training volume of knee flexion, there was no significant difference between alternative and successive training (CON phase of *p* = 0.198 and ECC phase *p* = 0.245). On the other hand, there was a significant difference in the relative change in the training volume of knee extension between alternative training and successive training (CON phase of *p* = 0.002 and ECC phase *p* = 0.021).

**Table 4:** Relative changes in muscle strength and training volume.

Measurement	Movement	Contraction	Alternative training (%)	Successive training (%)
Muscle strength (post/pre ratio)	Knee flexion	CON phase	93.3 ± 11.7	100.7 ± 13.0
		ECC phase	106.4 ± 13.0	105.3 ± 12.0
	Knee extension	CON phase	81.6 ± 20.6	89.7 ± 16.7
		ECC phase	104.8 ± 20.8	104.3 ± 16.7
Training volume (Set 3/Set 1 ratio)	Knee flexion	CON phase	88.4 ± 8.6	84.3 ± 13.7
		ECC phase	96.3 ± 11.7	91.5 ± 12.5
	Knee extension	CON phase	90.9 ± 10.2	83.3 ± 12.1*
		ECC phase	101.0 ± 20.3	88.2 ± 11.9*

CON, concentric contraction; ECC, eccentric contraction. Values were described as mean ± standard deviation.

* *p* < 0.05 vs. alternative training.

## 4 Discussion

In this study, isokinetic muscle strength in the knee flexion did not change in either the CON or ECC phases. On the other hand, in the knee extension, the CON phase decreased, but the ECC phase did not change. In addition, there was no significant difference in muscle strength between interventions. Kadota et al. (23) examined the effects of alternative training (paired-set training and super-set training) and successive training (traditional training) on the changes in the isokinetic muscle strength of CON in knee extension and knee flexion exercises. They reported that the muscle strength of CON in knee flexion decreased with alternative training but did not change with successive training. Furthermore, they reported that muscle strength decreased with both alternative and successive training in knee extension. These results are consistent with the results of this study regarding the muscle strength of the CON phase. Previous studies reported that untrained men required 120 s for full recovery of muscle strength (17, 27). Therefore, for knee flexions, it was possible that a decrease in the muscle strength of the CON phase after resistance training was recovered between the end of training and muscle strength measurement (at least 30 s of knee extension and 60 s of inter-set rest period). However, in the present study, since the muscle strength measurement for the knee extension was performed immediately after the end of training, the muscle strength of the knee extension could not have recovered. Previous studies reported that ECC has a lower energy cost and less fatiguability than CON (1, 4, 11, 12). In this study, the muscle strength of the ECC phase in knee flexion and extension did not change, and the results indicated less fatiguability in the ECC phase regardless of resistance training methods. Yoshida et al. (4) reported that there was no difference in the change in isometric strength before and after resistance training between the CON, ECC, and CON + ECC in 30 maximal isokinetic elbow flexion exercises. However, since ECC changes the optimal angle of maximum muscle strength (14), it is possible that the muscle fatigue of ECC cannot be accurately determined using isometric contractions. The present study examined the change in isokinetic muscle strength and showed that the change in the strength differed between the CON and ECC phases, with the ECC phase resulting in less fatiguability.

In this study, the training volume of the CON phase decreased with each set in both knee flexion and knee extension. In addition, the training volume of the ECC phase decreased in Set 3 in knee flexion, but it did not change in knee extension. These results indicated that the ECC phase had less fatiguability for training volume than the CON phase, regardless of the training methods, as with muscle strength measurements. The results of the present study showed a significant interaction between intervention and time for knee extension. The reason why the training method only affected knee extensions was unclear. However, it has been reported that when performing two resistance training programs (28, 29), the later exercise causes greater fatigue, so the order of knee flexion and knee extension exercises may have been related to the results of this study. In the present study, the training volume of knee extension did not change between sets in alternative training but decreased with each set in successive training. Previous review studies reported that a short-to-moderate duration of inter-set intervals of resistance training is sufficient for including gains in muscular strength for untrained individuals (15, 17). However, some previous studies reported greater gains in isokinetic strength when longer inter-set rest intervals were used (30, 31). Gentil et al. (32) randomized 34 untrained men to one of two groups: a group that followed a 1:3 work-to-rest ratio and a group that followed a 1:6 work-to-rest ratio. They reported no significant differences in muscle strength increase after 12 weeks of resistance training between the groups, indicating that a 1:3 work-to-rest ratio may be sufficient to elicit recovery between sets. In the present study, alternative training consisted of 30 s of resistance training and 150 s of rest period (30 s of antagonist training and two 60 s of inter-set rest period) for the agonist muscles, with a work-to-rest ratio of 1:5. On the other hand, successive training consisted of 30 s of resistance training and 60 s of inter-set rest intervals for the agonist muscles, with a work-to-rest ratio of 1:2. Therefore, it is suggested that alternative training may have sufficient rest periods to recover from muscle fatigue, resulting in maintaining high training volume.

In this study, there was no significant difference in the relative change in muscle strength (post/pre ratio) between alternative and successive training. On the other hand, for the relative change in the training volume of knee extension (Set 3/Set1 ratio), the CON and ECC phases of alternative training were significantly higher than those of successive training. These results suggested that differences in training methods may affect muscle fatigue during resistance training but not after training, regardless of whether it is the CON or ECC phases of CON + ECC. However, Shibata et al. (1) compared the effects of different inter-repetition periods (3 and 6 s) of a dumbbell arm curl exercise on failure to repetitions between ECC and CON. They found that failure to repetitions did not change in ECC but increased in CON (1.4–1.5 times greater in 6 s than in 3 s). Shibata et al. (1) investigated the difference in the inter-repetition rest period between CON and ECC for arm curls, while we examined the difference between alternative training and successive training in the CON phase and ECC phase of CON + ECC for knee flexion and knee extension exercises. The reasons for the difference between the results of this study and Shibata et al. (1) may be due to differences in muscles and training methods, and further study is needed.

This study has several limitations. This study was conducted on males who had no training experience. The effects of resistance training and inter-set rest intervals are affected by the sex (17, 33) and training experience (17, 24) of the individuals. This study compared the effects of two types of exercises (knee flexion and knee extension). Since resistance training often consists of multiple exercises and the order of the exercises could affect muscle fatigue, it is necessary to investigate the effects of order on three or more types of resistance.

## 5 Conclusions

This study examined the differences in muscle fatigue between the CON and ECC phases of CON + ECC with alternative and successive training of knee extension and knee flexion. The results of this study showed that both isokinetic muscle strength and training volume of the ECC phase had less fatiguability than those of the CON phase. Alternative training had a lower rate of change of decrease in training volume for knee extension than did successive training. The total training volume was higher in alternative training than that in successive training. These results indicated the difference in the training methods (alternative and successive training) did not affect the fatigue characteristics of CON and ECC.

## Data Availability

The original contributions presented in the study are included in the article/Supplementary Material, further inquiries can be directed to the corresponding author.
